# Application of Virtual and Augmented Reality Technology in Hip Surgery: Systematic Review

**DOI:** 10.2196/37599

**Published:** 2023-03-10

**Authors:** Peng Sun, Yao Zhao, Jie Men, Zhe-Ru Ma, Hao-Zhuo Jiang, Cheng-Yan Liu, Wei Feng

**Affiliations:** 1 Department of Bone and Joint Surgery, Orthopaedic Center The First Hospital of Jilin University Chang chun China; 2 Department of Bone and Joint Surgery Yantai Affiliated Hospital of Binzhou Medical University Yan tai China

**Keywords:** virtual reality, augmented reality, hip, pelvis, arthroplasty, mobile phone

## Abstract

**Background:**

Virtual and augmented reality (VAR) represents a combination of current state-of-the-art computer and imaging technologies and has the potential to be a revolutionary technology in many surgical fields. An increasing number of investigators have developed and applied VAR in hip-related surgery with the aim of using this technology to reduce hip surgery–related complications, improve surgical success rates, and reduce surgical risks. These technologies are beginning to be widely used in hip-related preoperative operation simulation and training, intraoperative navigation tools in the operating room, and postoperative rehabilitation.

**Objective:**

With the aim of reviewing the current status of virtual reality (VR) and augmented reality (AR) in hip-related surgery and summarizing its benefits, we discussed and briefly described the applicability, advantages, limitations, and future perspectives of VR and AR techniques in hip-related surgery, such as preoperative operation simulation and training; explored the possible future applications of AR in the operating room; and discussed the bright prospects of VR and AR technologies in postoperative rehabilitation after hip surgery.

**Methods:**

We searched the PubMed and Web of Science databases using the following key search terms: (“virtual reality” OR “augmented reality”) AND (“pelvis” OR “hip”). The literature on basic and clinical research related to the aforementioned key search terms, that is, studies evaluating the key factors, challenges, or problems of using of VAR technology in hip-related surgery, was collected.

**Results:**

A total of 40 studies and reports were included and classified into the following categories: total hip arthroplasty, hip resurfacing, femoral neck fracture, pelvic fracture, acetabular fracture, tumor, arthroscopy, and postoperative rehabilitation. Quality assessment could be performed in 30 studies. Among the clinical studies, there were 16 case series with an average score of 89 out of 100 points (89%) and 1 case report that scored 81 (SD 10.11) out of 100 points (81%) according to the Joanna Briggs Institute Critical Appraisal Checklist. Two cadaveric studies scored 85 of 100 points (85%) and 92 of 100 points (92%) according to the Quality Appraisal for Cadaveric Studies scale.

**Conclusions:**

VR and AR technologies hold great promise for hip-related surgeries, especially for preoperative operation simulation and training, feasibility applications in the operating room, and postoperative rehabilitation, and have the potential to assist orthopedic surgeons in operating more accurately and safely. More comparative studies are necessary, including studies focusing on clinical outcomes and cost-effectiveness.

## Introduction

### Background

Currently, there is consensus on the precise definitions of virtual reality (VR) or augmented reality (AR). Milgram et al [1] created a good taxonomy for AR devices and Muhanna [2] for VR devices. A VR environment is a completely synthetic world in which users can immerse themselves; this virtual world simulates the properties of the real world to a certain extent or surpasses the boundaries of physical reality by creating a world in which the physical laws that control the properties of gravity, time, and matter are no longer applicable [3]. The classification and explanation [4] of VR and AR are detailed in Multimedia Appendix 1. An increasing number of researchers are applying VR or AR techniques to hip-related procedures, such as preoperative operation simulation and training, certain applications in the operating room, and postoperative rehabilitation.

Virtual and augmented reality (VAR) technology is an emerging surgical technique that can enhance orthopedic surgeons’ competence by intuitively reinforcing medical information [5]. In this technique, users are presented a fully virtual environment through a monitor-based display [6], optical perspective systems, or video fluoroscopic systems [7]. Users can also visualize virtual content that is directly superimposed on reality, resulting in a high degree of flexibility [8]. Thus, VAR technology can help surgeons with not only surgical simulation but also intraoperative steps by showing the correct trajectory of movement for implant placement.

The average age at the time of hip fracture is 80 years, and the lifetime prevalence of hip fractures is 20% in women and 10% in men [9]. Osteoarthritis, which affects >240 million people worldwide, is the most common cause of restricted activity in adults and leads to joint dysfunction, pain, stiffness, limited function, and loss of valuable activities [10]. Most patients with hip fracture and end-stage osteoarthritis require surgical treatment, such as fracture reduction and internal fixation or arthroplasty [9,11,12]. During the course of postoperative rehabilitation, many complications inevitably occur, such as aseptic loosening, dislocation, and misalignment [13]. Indeed, the application of VAR technology has begun to bring about revolutionary changes in orthopedic surgery and training. Complex and delicate hip surgery procedures require an operator with extensive surgical experience, and surgeons lacking such experience face many potential problems in performing complex hip surgeries.

### Goal of This Study

Hence, the aim of this review was to determine the use of VR and AR techniques in hip-related preoperative operation simulator and training, postoperative rehabilitation, and feasibility applications in the operating room.

## Methods

The PRISMA (Preferred Reporting Items for Systematic Reviews and Meta-Analyses) guidelines (refer to Multimedia Appendix 2 for the full list) were followed during our literature search and the writing of our systematic review.

### Search Strategy and Selection Criteria

Two independent reviewers (PS and YZ) systematically searched the PubMed and Web of Science databases using the following key search terms: (“virtual reality” OR “augmented reality”) AND (“pelvis” OR “hip”). We believed that these databases would be appropriate because of the number of indexed journals and the coverage of related disciplines, such as bone and joint, clinical medicine, and computer science. When selecting academic databases, we also considered the flexibility of their search engines (for combining search terms) and the ability to export the results to a format accepted by the reference management software. Two investigators (PS and YZ) independently conducted literature screening based on the titles and abstracts of the studies. When the information in the titles and abstracts was not sufficient, we reviewed the full text to decide whether to include or exclude the studies. The reference lists of the included studies and existing reviews on the topic were screened to identify additional eligible studies. Any disagreement in the study selection process was resolved by a full discussion, and a third reviewer (JM) was consulted if a consensus could not be reached.

### Research Question

A review of the use of VAR technology in hip surgery is presented, with an emphasis on the evaluation of the necessity, advantages, and limitations of trials investigating the application of VAR technology in hip-related surgery. We also discuss the advantages of and potential barriers to VAR simulation training as compared with conventional training simulations and its future development and specifications.

### Selection Criteria

The literature inclusion criteria were as follows:

1. Basic and clinical research related to the aforementioned key search terms, that is, studies evaluating the key factors, challenges, or problems of VAR technology in hip-related surgery
2. The study type was a monograph, paper, guide, or review

The exclusion criteria were as follows:

1. Studies that did not focus on hip or pelvic surgery
2. Studies that did not have human participants
3. Studies that did not focus on VR or AR
4. Studies with low-quality or low-level evidence
5. Studies that did not resolve any of the aforementioned issues

### Data Extraction and Quality Assessment

A predesigned extraction form was used to extract the data. The extracted data included the name of the first author, type classification, visualization, preoperative simulation and training or intraoperative applications and postoperative rehabilitation, nature of the study, patients or diseases, intervention procedures, comparison measures, and outcomes. Disagreements in data extraction were resolved through discussions between the 2 investigators (PS and YZ), and a third reviewer (ZRM) was consulted if necessary. The quality of all the studies including real patients was then assessed using the Joanna Briggs Institute Critical Appraisal Checklist [14]. A scoring system was used to qualify the studies: studies that answered yes to a question from the checklist were scored 2, studies whose answers to the checklist question were not clear were scored 1, and studies that answered no to the checklist question were scored 0 [15].

## Results

### Literature Search

**Figure 1:**
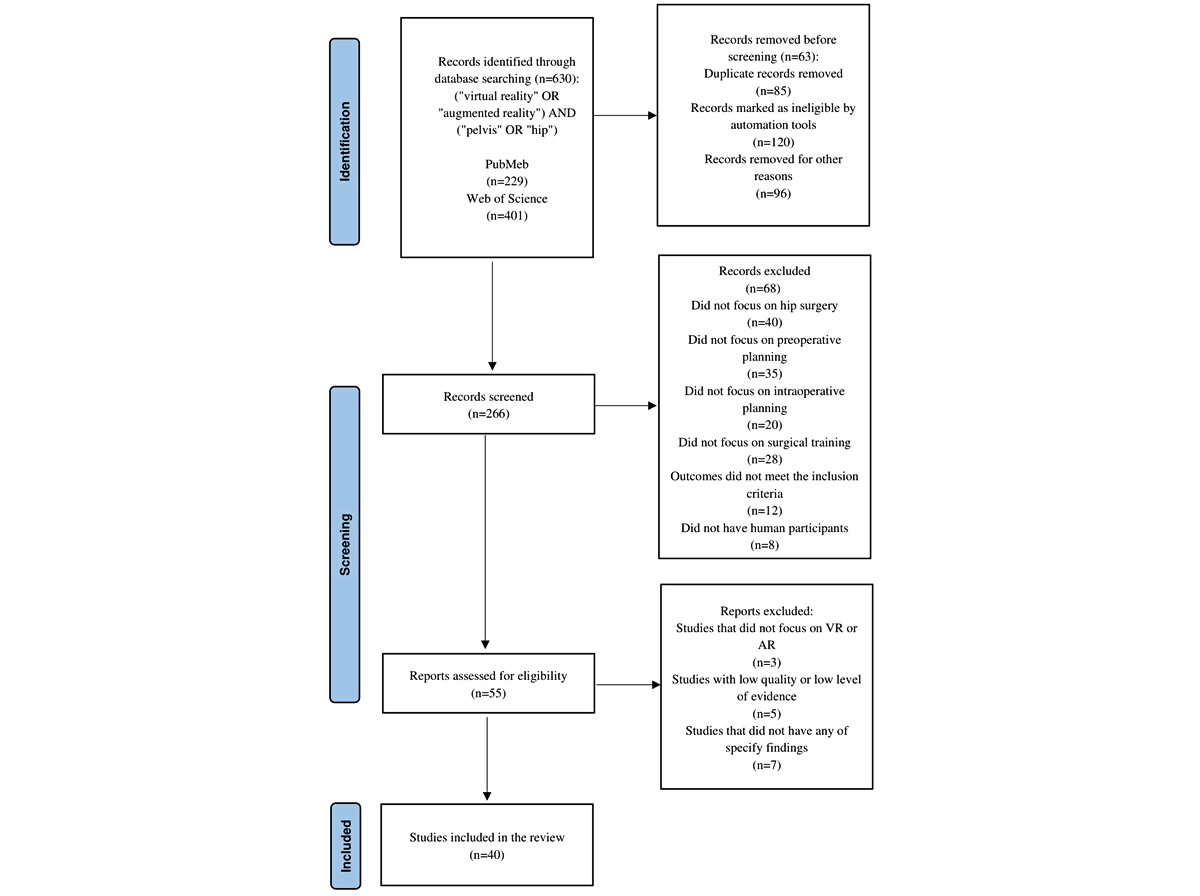
PRISMA (Preferred Reporting Items for Systematic Reviews and Meta-Analyses) 2020 flow diagram adapted for this study. AR: augmented reality; VR: virtual reality.

Using the aforementioned search strategies, we retrieved >630 potentially relevant papers published between 1992 and 2022. We identified a total of 22 studies on the application of VR in hip surgery (namely 9 studies on primary total hip arthroplasty, 41%; 4 on hip fracture, 18%; 3 on pelvic fracture, 14%; 1 on hip tumor, 5%; 3 on hip arthroscopy, 14%; and 2 on postoperative rehabilitation after total hip arthroplasty, 9%); 13 studies on the application of AR in hip surgery (namely 4 studies on acetabular cup placement during total hip arthroplasty, 31%; 1 on hip resurfacing, 8%; 3 on pelvic fracture, 23%; 1 on acetabular fracture, 8%; 1 on pelvic and acetabular fractures, 8%; 1 on femoral neck fracture, 8%; and 2 on hip tumor, 15%); and 5 studies on emerging devices using VR and AR technologies. A schematic stepwise algorithm for the search strategy is shown in Figure 1.

The first screening step was to eliminate duplicate studies using a software, which resulted in the exclusion of 364 papers. The second step was to exclude studies according to the exclusion criteria (n=211). We then assessed the eligibility of the studies based on their quality and excluded substandard studies (n=15). Finally, we included 40 studies.

### Study Characteristics

The included articles were published between 1992 and 2022. Each of these articles covered the implementation of VAR in a different procedure. The classification of VAR devices, visualization, nature of the study, patients or diseases, intervention, comparison, and outcome characteristics are presented in Tables 1 and 2.

**Table 1:** Summary of the included studies—author name, study type, visualization, application, and nature.

Study	Type	Visualization	Application	Nature
Pransky [16]	VR^a^	Orthodoc + preplanning workstation + 3D image + CT^b^	Preoperative simulation and training	Concept study
Sato et al [17]	VR	CAF^c^ system + pelvis and femur system + 3D model + CT	Preoperative simulation Intraoperative application	Clinical trial
Digioia et al [18]	VR	HipNav + 3D software + CT	Preoperative simulation Intraoperative application	Clinical trial
Takada et al [19]	VR	HipAlign and manual goniometer + x-rays or fluoroscopy	Preoperative simulation Intraoperative application	RCT^d^
Ruikar [20]	VR	2D game	Preoperative simulation and training	N/A^e^
Barrack et al [21]	VR	MicroScribeTm 3DX digitizer + LightWave 3DTm software	Preoperative simulation and training	Concept study
Krushell et al [22]	VR	3D protractor + Sawbones pelvis + modular THA^f^ system	Preoperative simulation and training	Concept study
Kummer et al [23]	VR	Femoral components + Sawbones hemipelvis + goniometer	Preoperative simulation and training	Clinical trial
Scifert et al [24]	VR	Blueprints; CAD^g^ models + Duraloc	Preoperative simulation and training	Concept study
Cimerman et al [25]	VR	SQ^h^ Pelvis software + CT in DICOM	Preoperative simulation and training	Case series
Brouwers et al [26]	VR	3D printing + VR headset + hemipelvis	Preoperative simulation and training	Clinical trial
Tonetti et al [27]	VR	CT images + an ultrasound registration + specimen pelvis	Preoperative simulation and training	Clinical trial
Blyth et al [28]	VR	Bonedoc DHS^i^ simulator	Preoperative simulation and training	RCT
Tasi et al [29]	VR	Volume-based orthopedic surgery simulator	Preoperative simulation and training	Clinical trial
Rambani et al [30]	VR	CAOS^j^ + computer-screen and x-ray images	Preoperative simulation and training	Clinical trial
Racy et al [31]	VR	3D virtual environment + haptic + printed drill handle + VR headset	Preoperative simulation and training	Clinical trial
Handels et al [32]	VR	VR + 3D medical objects + CT or MR^k^	Preoperative simulation and training	Clinical trial
Khanduja et al [33]	VR	VR simulator	Preoperative simulation and training	RCT
Bishop et al [34]	VR	Hip arthroscopy virtual simulator using ASSET^l^	Preoperative simulation and training	RCT
Bartlett et al [35]	VR	Hip arthroscopy simulator	Preoperative simulation and training	RCT
Fascio et al [36]	VR	VRRS^m^: wearable sensors	Postoperative rehabilitation	RCT
Zavala-González et al [37]	VR	Nintendo Wii game	Postoperative rehabilitation	RCT
Alexander et al [38]	AR^n^	AR environment + RGBD^o^ camera	Preoperative simulation and training	RCT
Ogawa et al [39]	AR	A goniometer and AR-HIP^p^ system	Intraoperative applications	RCT
Fotouhi et al [40]	AR	Two C-arm x-ray images + 3D AR visualization + real-time RGBD data overlay	Preoperative simulation and training	Clinical trial
Logishetty et al [41]	AR	AR headsets with MicronTracker and HoloLens hardware	Preoperative simulation and training	RCT
Liu et al [42]	AR	AR-based navigation system + depth sensing + HoloLens	Preoperative simulation and training	Concept study
Befrui et al [43]	AR	C-arm + RGBD camera	Preoperative simulation and training	Clinical trial
Wang et al [44]	AR	AR-based navigation system	Intraoperative applications	Pilot study
Chen et al [45]	AR	AR-SNS^q^ + HMD^r^	Preoperative simulation and training	Concept study
Fornaro et al [46]	AR	Patient-specific bone model from preoperative CT scans + visuo-haptic feedback	Preoperative simulation and training	Clinical trial
Shen et al [47]	AR	AR-aided implant design and contouring system	Preoperative simulation and training	Clinical trial
van Duren et al [48]	AR	A digital fluoroscopic imaging simulator using orthogonal cameras	Preoperative simulation and training	Concept study
García-Sevilla et al [49]	AR	PSIs^s^ system using a smartphone and the HoloLens 2	Preoperative simulation and training	Clinical trial
Postl et al [50]	AR	Navigation system and K-wires as guidance for the oscillating saw	Preoperative simulation and training	Cadaver study

^a^VR: virtual reality.

^b^CT: computed tomography.

^c^CAF: combined acetabular and femur.

^d^RCT: randomized controlled trial.

^e^N/A: not applicable.

^f^THA: total hip arthroplasty.

^g^CAD: computer-aided design.

^h^SQ: standard quality.

^i^DHS: dynamic hip screw.

^j^CAOS: computer-assisted orthopedic training system.

^k^MR: magnetic resonance.

^l^ASSET: arthroscopic surgery skill evaluation tool.

^m^VRRS: virtual reality rehabilitation system.

^n^AR: augmented reality.

^o^RGBD: red-green-blue-depth.

^p^AR-HIP: augmented reality hip.

^q^SNS: surgical navigation system.

^r^HMD: head-mounted display.

^s^PSI: patient-specific instrument.

**Table 2:** Summary of the included studies—patient or disease, intervention, comparison, and outcome.

Patient or disease	Intervention	Comparison	Outcome
Osteoarthritis and ONFH^a^	THA^b^	N/A^c^	N/A
Osteoarthritis and ONFH	THA	N/A	Cup position accuracy Cup orientation Limb length
Osteoarthritis and ONFH	THA	N/A	Range of motion testing Cup orientation
Osteoarthritis and ONFH	THA	HipAlign Manual goniometer	Cup orientation (*P*<.01) Cup inclination (*P*<.01)
Osteoarthritis and ONFH	THA	N/A	Cup size Cup position Femoral stem orientation
Osteoarthritis and ONFH	THA	N/A	Cup orientation
Osteoarthritis and ONFH	THA	N/A	Acetabular component motion
Osteoarthritis and ONFH	THA	N/A	Cup orientation
Osteoarthritis and ONFH	THA	N/A	Total hip dislocation resistance
Pelvic fracture	Screws fixation	N/A	Computerized modules assessment
Pelvic fracture	Classify acetabular fractures	N/A	Fractures classification (*P*>.99)
Pelvic fracture	Percutaneous iliosacral screw placement	N/A	Number of x-rays
Hip fracture	Dynamic hip screw	Medical group Trainee group Advanced train group	Reduction Incision length Misplaced drill holes Screw placement X-rays Surgical time (*P*=.01)
Hip fracture	Hip fracture + plate surgery	N/A	Drilling force Torque computation
Hip fracture	Dynamic hip screw + fracture fixation	12 orthopedic officers performed dynamic hip screw fixation	Time Accuracy of fixation The number of exposures (*P*=.04)
Hip fracture	Proximal guidewire entry and distal locking	Orthopedic specialist trainees Consultants	X-rays Authenticity and content validity
Bone tumor	Bone tumor surgery	N/A	Resection planes Patient’s anatomy
Soft-tissue and muscle injuries and osteoarthritis	A task testing basic probe examination of the joint	Novice surgeons; n=10 Experienced surgeons; n=9	Time (*P*<.001) Collisions with soft tissue (*P*=.001) Collisions with bone (*P*=.002) Distance traveled (*P*=.02)
Soft-tissue and muscle injuries and osteoarthritis	Diagnostic arthroscopy	30 participants (23 males and 7 females)	ASSET^d^ scores (*P*=.04)
Soft-tissue and muscle injuries and osteoarthritis	Performed diagnostic supine hip arthroscopies	Faculty members; n=7 Orthopedic residents; n=18	Face validity questionnaire responses
Osteoarthritis ONFH and periarticular fracture of hip	Perform a daily home exercise program	VRRS^e^; n=21 Control; n=22	Hip disability Level of independence Degree of global perceived effect (*P*<.001)
Osteoarthritis, ONFH, and periarticular fracture of hip	Physical therapy of patients who underwent THA	Physiotherapy treatment Nintendo Wii	WOMAC^f^ questionnaire Berg Balance Scale Six-minute walk distance test Weight load
Osteoarthritis and ONFH	Navigation for acetabular cup placement	8 orthopedic surgery trainees completed component placement	Cup inclination (*P*=.01) Cup anteversion (*P*=.02) Time (*P*=.008) SUS^g^; STLI^h^ (*P*=.04) Radiation dose (*P*=.48)
Osteoarthritis and ONFH	Acetabular cup placement	Goniometer AR-HIP^i^ system	Cup anteversion (*P*<.001) Cup inclination (*P*=.13)
Osteoarthritis and ONFH	Navigation for acetabular cup placement	N/A	Errors in translation Anteversion Abduction Radiation
Osteoarthritis and ONFH	Acetabular cup placement	Group that was trained using AR^j^ Group that received one-on-one training	AR guidance errors in orientation (*P*<.001) Assessment improvement (*P*<.001)
Osteoarthritis, ONFH, and periarticular fracture of hip	Drilling of guide hole	N/A	Errors in position Direction of the experiment
Pelvic fracture	K-wire placement	N/A	Simulated clinical efficiency K-wire placement evaluation
Pelvic fracture	Sacroiliac screw insertion	N/A	Accuracy evaluation Screw positions and the deviations
Pelvic fracture	Percutaneous sacroiliac screw implantation	N/A	Accuracy verification Mean distance and angular errors
Acetabular fracture	Acetabular fracture reconstruction	N/A	Postoperative CT^k^ landmark
Pelvic and acetabular fractures	Unilateral pelvic and acetabular fracture reduction	N/A	Reliability based on interobserver reproducibility
Femoral neck fracture	Guidewire insertion in DHS^l^	N/A	Accuracy of the calculated TAD^m^
Bone tumor	Pelvic tumor resection	AR guidance resections Freehand resections	Osteotomy deviations Shape and location of PSIs^n^
Bone tumor	Supra-acetabular pelvic tumor resections	Computer-aided resections Freehand resections	Deviation of the navigated osteotomies (*P*<.001)

^a^ONFH: osteonecrosis of the femoral head.

^b^THA: total hip arthroplasty.

^c^N/A: not applicable.

^d^ASSET: arthroscopic surgery skill evaluation tool.

^e^VRRS: virtual reality rehabilitation system.

^f^WOMAC: Western Ontario and McMaster universities arthritis index.

^g^SUS: System Usability Scale.

^h^STLI: surgical task load index.

^i^AR-HIP: augmented reality hip.

^j^AR: augmented reality.

^k^CT: computed tomography.

^l^DHSl: dynamic hip screw.

^m^TAD: tip-apex distance.

^n^PSI: patient-specific instrument.

### Quality Assessment

The quality assessment process could be performed in only 73% (30/40) of the studies, in which either human study populations or cadavers were evaluated. As such, of 40 studies, 5 (12%) studies included patients, and 2 (5%) described cadaveric results. Among the clinical studies, there were 2% (1/40) of case series with an average score of 85 out of 100 points (85%) and 16 (16%) case series with an average score of 89 (SD 10.11) out of 100 points (89%) according to the Joanna Briggs Institute Critical Appraisal Checklist (refer to Multimedia Appendix 3 [17-19,21,25,26,28,30,31,33,34,36,38,39,41,45,49] for the full list). The 2 cadaveric studies also scored high based on Quality Appraisal for Cadaveric Studies scale, with average scores of 85 out of 100 points (85%) and 92 out of 100 points (92%) (refer to Multimedia Appendix 4 for the full list [44,50]).

### Virtual Reality

#### Total Hip Arthroplasty

The ROBODOC [16] is a robotic system designed for human hip replacement surgery. The first human hip replacement surgery using the ROBODOC system was performed in a man aged 64 years. This tool allowed the orthopedic surgeons to accurately examine the patient’s skeleton and develop preoperative plans before total hip replacement surgery. As the authors used an individualized approach, they were able to reduce postoperative complications to a great extent, leading to good patient satisfaction [51]. It is difficult for new surgeons to judge the direction of femoral osteotomy, the location in which to look for the true acetabulum where the prosthesis will be installed, and the exposure of the anatomical safety margin. Sato et al [17] performed a preoperative simulation of total hip replacement surgery using a 3D preoperative hip-implantation planning tool. The simulation system they used helps select optimal surgical parameters, such as acetabulum cup size and position, and femoral stem orientation.

The combination of advanced VR simulation technology and 3D visualization allows users to quickly and intuitively understand the results of implant placement in hip arthroplasty. Interactive feedback and intuitive control mechanisms help identify the optimal implant location for a given patient’s anatomy. The HipNav system developed by Digioia et al [18] remains the most comprehensive total hip replacement planner. This system represents the first clinical application of the concept of hip navigation. The preoperative 3D reconstruction simulation software of the HipNav system allows surgeons to simulate the position of the acetabular component within the pelvis based on the preoperative computed tomography (CT) images. HipNav also includes hip kinematic models and tools for predicting the femoral range of motion and skeletal motion as well as optimal parameter calibration based on implant placement. The feedback provided by the simulator can assist surgeons in determining the optimal, patient-specific placement of acetabular implants. The HipAlign navigation system was also designed based on the concept of hip navigation. This portable system combines the accuracy of image-free computer-based navigation systems with the convenience of traditional alignment techniques. Takada et al [19] prospectively assessed acetabular cup positioning using the portable image-free navigation system HipAlign and a manual goniometer during a procedure that was performed through a supine anterolateral approach; see Takada et al [19] for an image of a navigation sensor for a cup impactor. The absolute error of the difference between the CT-measured acetabular cup angle and HipAlign-measured acetabular cup angle was estimated. The authors reported that the HipAlign measurement was closer to the result obtained using postoperative CT and that HipAlign can be used to assess acetabular cup orientation during surgery. Notably, this navigation system can avoid incorrect acetabular cup anteversion in the supine position during total hip arthroplasty via a minimally invasive anterolateral approach.

Game-based surgical training is emerging as an educational tool for hip replacement and resurfacing because of the complexity of the spatial angulation involved and the lack of clinical theoretical studies on the location of the acetabular cup during acetabular cup placement. Edheads [20], a company that aims to inspire students to pursue science, technology, engineering, and mathematics careers, has designed interactive games that teach total hip replacement and resurfacing to children so that they can acquire knowledge through interactive experiences.

Acetabular subluxation often occurs after femoral neck impingement. Other potential adverse effects of early impingement include accelerated polyethylene wear, acetabular component loosening, and linear dislocation. Owing to the numerous potential adverse effects of early component impingement, it is advantageous to clarify the range of component motion before the components come into contact with each other. Kummer et al [21] designed femoral and acetabular components and then digitized them using a MicroScribe 3DX (Immersion) digitizer and light wave VeriSketch 3D (Gravity Sketch) software. The authors innovatively used VR software and computer animation to determine the effects of component positioning and prosthesis design on the range of motion before impingement after total hip arthroplasty. However, they did not model the effects of femoral acetabular impingement on the pelvis, soft tissues, or osteophytes. Another limitation of their study is that other causes of instability, such as soft-tissue imbalance, muscle weakness, and low patient compliance, were not considered. The primary purpose of the total hip arthroplasty design is to maximize the range of motion and stability. Krushell et al [22] used a simple goniometer to evaluate the effect of component design parameters on the range of motion after total hip arthroplasty and determined that a skirted head and certain types of lip liners reduced the range of motion after total hip arthroplasty. Kummer et al [23] implanted 2 typical cemented femoral stems in a Sawbones model and measured the range of motion as the component position changed. Their results showed that 35° to 45° inclination and 0° to 10° anteversion were optimal. They further demonstrated that anteversion >20° limits internal and external hip rotation. Thus, the authors reported that while modifications to the acetabular cup design can enhance joint mobility, the design and position of the femoral implant also play a role in determining the range of rotation. The reason for the increased likelihood of dislocation is unknown, but soft-tissue stretching or injury that may occur during early dislocation may be a key factor. In patients with recurrent dislocations, the additional range of motion may increase instability owing to excessive injury to the acetabular liner rim as a result of impingement. Scifert et al [24] used 3D finite element analysis to evaluate intraoperative implant placement variables and parameter settings and the ability of the implant to resist posterior dislocation. A single activity, specifically, leg crossing in an upright sitting position, was chosen to determine the possibility of dislocation. The authors found that increasing the forward component and decreasing the abduction component improved the range of motion and peak resistance moment in posterior dislocations.

#### Fracture

##### Pelvic Fracture

The purpose of the surgical treatment of pelvic fracture is to maintain the anatomical shape of the pelvis and restore its biomechanical characteristics. When reconstructing acetabular fractures, the basic principles of anatomical reconstruction, stable fixation of the articular surface, and immediate postoperative exercises should be observed. Cimerman et al [25] introduced a computer program for virtual surgery experiments of pelvic and acetabular fractures based on real fracture data; see Cimerman et al [25] for an image of an advanced computerized planning module. Using the 3D viewing tool, the surgeon can build a virtual model of the pelvic fracture. This case study demonstrates the possibility of virtual simulated surgery. The computer program is an easy-to-use application program with great potential for application in clinical practice, teaching, and research. Acetabular fractures are difficult to classify because of the complex 3D anatomical structure of the pelvis; 3D printing is helpful for understanding and reliably classifying acetabular fractures, and 3D VR may have similar benefits. Brouwers et al [26] hypothesized that 3D VR is equivalent to 3D printing in terms of understanding acetabular fracture patterns. They believe that VR can also provide a “realistic” 3D view. The effectiveness of 3D VR and 3D printing in promoting fracture classification was evaluated, and the authors found that 3D VR was less effective than 3D printed models of acetabular fractures. In addition, current 3D VR technology is not suitable for intraoperative use. In the future, advances in VR technology may enable its intraoperative use for the treatment of acetabular fractures.

Iliosacral screw placement is a useful technique for the fixation of posterior pelvic ring injuries. If the pelvic ring is broken, percutaneous iliosacral screw fixation can be performed in the supine position using computer imaging techniques [52-54]. This ensures early fixation for patients with multiple traumas and significantly reduces the risk of bleeding or infectious complications at the surgical site [55]. Tonetti et al [27] aimed to evaluate the educational efficiency of a fluoroscopically guided path simulator for the percutaneous screw fixation of the sacroiliac joint. They evaluated the accuracy of 23 surgeons inserting guidewires according to predetermined procedures in human cadaveric experiments. VR simulation of iliosacral screw insertion was found to reduce the need for intraoperative photography when positioning the guidewire in human cadavers. Novice surgeons who have good anatomical knowledge of the lumbosacral joint but are not used to surgery are the ones who can benefit the most from this valuable tool.

##### Hip Fracture

The objective structured assessment of surgical skills provides a method for assessing the technical skills of students [56]. Although this assessment is considered essential, it is rarely conducted because of the high cost, personnel requirements, lack of objectivity of labeling, and possible surgery-related issues [57]. VR has the potential to help overcome some of these issues [58]. Blyth et al [28] recently developed the Bonedoc, a VR simulator for the screw and plate fixation of hip fractures, to solve some of these problems. The Bonedoc simulator integrates all related tasks of hip fracture fixation, from fracture reduction, skin incision, and guidewire placement to final plate-and-screw placement. It automatically calculates accurate positions for fracture reduction and lag screw placement and other objective data. The aforementioned study showed that the Bonedoc simulator could distinguish novice surgeons from surgical trainees; however, its ability to discriminate between basic and advanced trainees was poor. Many studies have used perceptible tactile devices with simulators. Tsai et al [29] introduced a simulator with tactile capabilities to simulate the process of drilling the hip joint during screw and plate surgery and to locate trochanteric hip fractures. Simulation of the drilling process can also be used for surgical training. It is not clear whether a vibration sensation was included in this simulator. Owing to the 1-kHz technical limitation of the response frequency of the Geomagic Touch X5 tactile device, higher-frequency vibrotactile cues may not be accurately replicated by the simulator. In addition, the simulator may not account for the weight of the surgical drill in the trainee’s hands. Surgical training is severely affected by the challenges of reduced training opportunities, shorter working hours, and economic pressure. There is an increasing need to use training systems for training the psychomotor skills of surgical interns. Rambani et al [30] developed a training system for fracture fixation and validated its effectiveness in a cohort of junior orthopedic trainees; see Rambani et al [30] for an image of a computer-assisted orthopedic training system. The computer navigation training system was a good training tool for young orthopedic students. The system could be used to complement the training provided in the operating room. The trainees could be in a *threat-free and unhurried environment*. The system may be used in other orthopedic operations to learn technical skills and ensure the smooth upgrading of task complexity so as to improve the trainees’ performance of the actual operation in the operating room.

From low-cost task trainers to complex VR solutions, the development of simulators in various surgical specialties has increased substantially. Recent technological advances have enabled the creation of realistic VR environments with tactile feedback. Synthetic bone simulators are the most commonly used simulators in orthopedic training but have considerable limitations: they usually do not simulate soft tissue or use real patient positioning [51]. Racy et al [31] created a VR femoral nail simulator that combines an immersive VR environment with tactile and full image–intensifier functions and then conducted a validation study to evaluate its educational value; see Racy et al [31] for an image of a 3D virtual environment. By integrating multiple aspects of surgical practice into a single device, the authors aimed to improve the participants’ immersion and the tool’s educational value. Thus far, their work has focused on technical skills and shown good authenticity, content, and structural validity.

#### Tumor

The central step of the planning procedure for hip tumor surgery is to place the cutting plane in the hip bone, which largely depends on the location of the tumor. Segmentation of the tumor and bone in magnetic resonance and CT data and fusion of magnetic resonance and CT image sequences are necessary to visualize the location of the tumor in the hip. Handels et al [32] introduced a VIRTOPS software system for the virtual simulation of hip surgery. This system was used to simulate the reconstruction of the hip joint using a prosthesis during hemipelvis replacement and to support the personalized design of modular prostheses with strong anatomical adaptability in bone tumor surgery. The VIRTOPS system can realize complete virtual planning and prosthesis reconstruction of hip joint as well as the optimal placement and design of the prosthesis. It provides a general platform for the 3D planning and simulation of plastic surgery. It can also be used to simulate the implantation of a prefabricated prosthesis and study its match with a single pelvis.

### Hip Arthroscopy

#### Overview

With increasing applications in diagnosis and treatment, hip arthroscopy is one of the most rapidly evolving areas in modern surgery [59,60]. The studies assessing the effectiveness of VR simulators for hip arthroscopy are presented in Table 3. The ball-and-socket nature of the joint, the thickness of the joint capsule, and soft-tissue envelope make hip arthroscopy a technically demanding procedure with a steep learning curve [59]. Khanduja et al [33] tested the construct validity of the hip diagnostics module of a VR hip arthroscopy simulator; see Khanduja et al [33] for an image of a simulated arthroscopic examination. In their study, 19 orthopedic surgeons performed a simulated arthroscopic examination of a healthy hip joint using a 70° arthroscope and a supine patient position. Significant differences were observed in the average time required for basic visualization tasks, number of soft-tissue collisions, number of bone collisions, and camera-tissue contact time. No significant between-group differences were observed in any of the measurements during the basic probe examination. The use of low- and high-fidelity surgical simulation tools as auxiliary means of clinical contact in orthopedic training is increasing.

**Table 3:** Studies assessing the effectiveness of virtual reality (VR) simulators for hip arthroscopy.

Study	Simulation task	Participants	Outcomes assessed	Results and conclusions
Khanduja et al [33]	Hip arthroscopy: basic navigation and probe examination	10 novice surgeons (<250 independent arthroscopies) and 9 experienced surgeons (≥250 independent arthroscopies)	Time required to complete the task, number of soft-tissue collisions, number of skeletal collisions, camera-tissue contact time, distance achievable by the arthroscope, and femoral head scratch length	Significant differences in the average time required for basic visualization tasks, number of soft-tissue collisions, number of bone collisions, and camera-tissue contact time. No significant between-group differences in any of the measurements during the basic probe examination.
Bishop et al [34]	To complete a diagnostic arthroscopy and a loose body retrieval simulation	12 novices (medical students, PGY^a^1-2), 5 intermediate trainees (PGY3-4), 9 senior trainees (PGY5 and fellows), and 4 attending faculty	Higher ASSET^b^ scores, number of loose bodies retrieved, operation time, camera path and grasper path lengths, and the percentage of cartilage injury	VirtaMed Hip arthroscopy simulator has good structural validity and reliability in simulator-based indicators and ASSET scores. The performance of hip arthroscopic simulation could be more comprehensively evaluated using simulator indexes and ASSET than using either type of index alone.
Bartlett et al [35]	To test the face validity of the hip diagnostics module	7 faculty members and 18 orthopedic residents	Face validity questionnaire answers	The VR hip arthroscopy simulator has fidelity to establish its facial effectiveness. The simulator has enough authenticity to inculcate basic arthroscopic skills, which supports its use in orthopedic surgery training.

^a^PGY: postgraduate year.

^b^ASSET: arthroscopic surgery skill evaluation tool.

The increase in the working time of orthopedic trainees and the priority given by the global modern medical system to patient safety have led to a decline in the surgical autonomy of surgical trainees. Therefore, training courses that emphasize the simulation of surgical skills outside the operating room have steadily developed. Bishop et al [34] assessed the structural validity and interobserver reliability of a virtual simulator of hip arthroscopy using the global rating scale of the Arthroscopic Surgical Skill Evaluation Tool. A total of 30 participants (23 men and 7 women) completed 2 diagnostic arthroscopic simulations and a loose body retrieval simulation at least 1 week apart on the VirtaMed ArthroS Hip simulator. The authors confirmed that the VirtaMed ArthroS simulator has good structural validity and reliability in terms of simulator-based indicators and Arthroscopic Surgical Skill Evaluation Tool scores. Over the past decade, numerous researchers have studied the application of VR simulations in surgical education. The high technical requirements of hip arthroscopy coupled with a reduction in the operation time of students have led to a steep learning curve in modern orthopedic surgery [61]. Bartlett et al [35] tested the face validity of the hip joint diagnosis module of a VR hip arthroscopy simulator; they performed diagnostic supine hip arthroscopies of a virtual healthy hip joint using a 70° arthroscope. The hip arthroscopic diagnostic module was found to have an acceptable level of authenticity in all areas, except for the tactile feedback received from the soft tissue. The simulator had sufficient authenticity to inculcate basic arthroscopic skills and support its use in orthopedic surgery training.

#### Postoperative Rehabilitation

Early rehabilitation after total hip arthroplasty is very important for proper functional recovery [62]. However, outpatient access to rehabilitation services after surgery may be limited by social, physical, or environmental barriers. Telerehabilitation can help overcome these problems by allowing treatment to be performed directly at the patient’s home [63]. The benefits of early VR-based home rehabilitation after total hip arthroplasty have not been evaluated in detail. Fascio et al [36] compared the efficacy of early rehabilitation using a VR rehabilitation system with that of conventional rehabilitation in improving functional outcomes after total hip replacement. The conventional rehabilitation program and VR-based home rehabilitation program resulted in similar improvements in functional outcomes after total hip replacement. The application of VR simulation can provide new possibilities for rehabilitation services.

Postoperative physical therapy for patients who underwent total hip arthroplasty is generally considered to be effective in reducing pain and disability. However, after hip replacement, the muscle strength, postural stability, balance, and gait speed of patients will affect the function and performance of activities of daily living. In addition, it has been reported that the weight load between the lower limbs changes, which increases the strength capacity of the hip joint–stabilizing muscle tissue and adversely affects the function of these patients. Zavala-González et al [37] explored the clinical effects of applying VR technology in the physical therapy of patients who underwent total hip arthroplasty by means of the Nintendo Wii game console and its Wii Balance Board. In the short term, the addition of VR by means of Nintendo Wii and its Wii Balance Board platform resulted in statistically significant differences in the functioning of patients who underwent total hip replacement, but these differences were not clinically important. However, this finding has clinical importance. It shows that the application of VR in physical therapy can improve the clinical effects of rehabilitation in these patients.

### Augmented Reality

#### Total Hip Arthroplasty

##### Acetabular Cup Placement

Given the anticipated increase in the longevity and activity level of patients after total hip arthroplasty, the longevity of the prosthetic components used is critical. Accurate acetabular component positioning is essential to ensure good outcomes. Inaccurate placement may result in impingement, malposition, accelerated wear, loosening of components, and the need for modification. Alexander et al [38] used a radiopaque foam pelvis to simulate component placement; see Alexander et al [38] for an image of component placement using a radiopaque foam pelvis. Cone-beam CT data and optical data from a red-green-blue-depth camera were co-registered to create an AR environment, and the usability of the novel 3D AR guidance system was compared with standard fluoroscopy-guided acetabular component placement. The results showed that the AR technique was more accurate in terms of anteversion and inclination during the placement of the acetabular component than the standard fluoroscopic technique. The AR technique was also faster, without increasing the radiation dose. Similarly, the application of AR technology in acetabular cup placement during total hip arthroplasty was studied by Ogawa et al [39] They developed an acetabular cup placement device, called the AR-hip system, using AR technology. The AR-hip system allows the surgeon to view images of the acetabular cup superimposed on the operative field by means of a smartphone. The smartphone also shows the angle of placement of the acetabular cup. Compared with conventional technology, the AR-hip system provided a more accurate intraoperative acetabular cup placement angle. Although the AR-hip system can display acetabular cup images superimposed on the operating field, the value of this system as a navigation tool is unclear. The time to repeat surgery is influenced by implant wear, which is related to the physical characteristics of the implant as well as the position of the acetabular component. Conversely, appropriate implant placement can restore hip anatomy and biomechanics and reduce the risk of dislocation, impingement, loosening, and limb length discrepancy, thus reducing implant wear and revision rates. An easy-to-use intraoperative component planning system based on 2 C-arm x-ray images combined with 3D AR visualization was presented by Fotouhi et al [40]. This system simplifies the placement of the impactor and acetabular cup by providing real-time red-green-blue-depth data superimposition. The system also helps reduce radiation, operation time, and frustration and increases the efficiency and accuracy of the placement of the acetabular component. Ultimately, this approach may help reduce the rate of revision surgery in patients with hip disease.

For joint replacement, simulation training is typically performed on dry bones or cadavers. The former has low fidelity, whereas the latter has a high cost and requires an internal structure. Neither can objectively measure technology or 3D orientation skills [64]. A MicronTracker camera was integrated with the HoloLens AR headset system by Logishetty et al [41] to develop an enhanced AR headset that can track the position and orientation of the implant relative to the pelvis; see Logishetty et al [41] for an image of an enhanced AR headset. The platform can use real instruments and provide real-time feedback. Therefore, AR is considered a feasible and valuable training tool and can be used as an auxiliary tool for expert guidance in the operating room. Although there was no difference in accuracy between the group trained with AR and the group trained by expert surgeons, the authors believed that the MicronTracker camera and HoloLens AR headset system may be useful in education.

##### Hip Resurfacing

**Figure 2:**
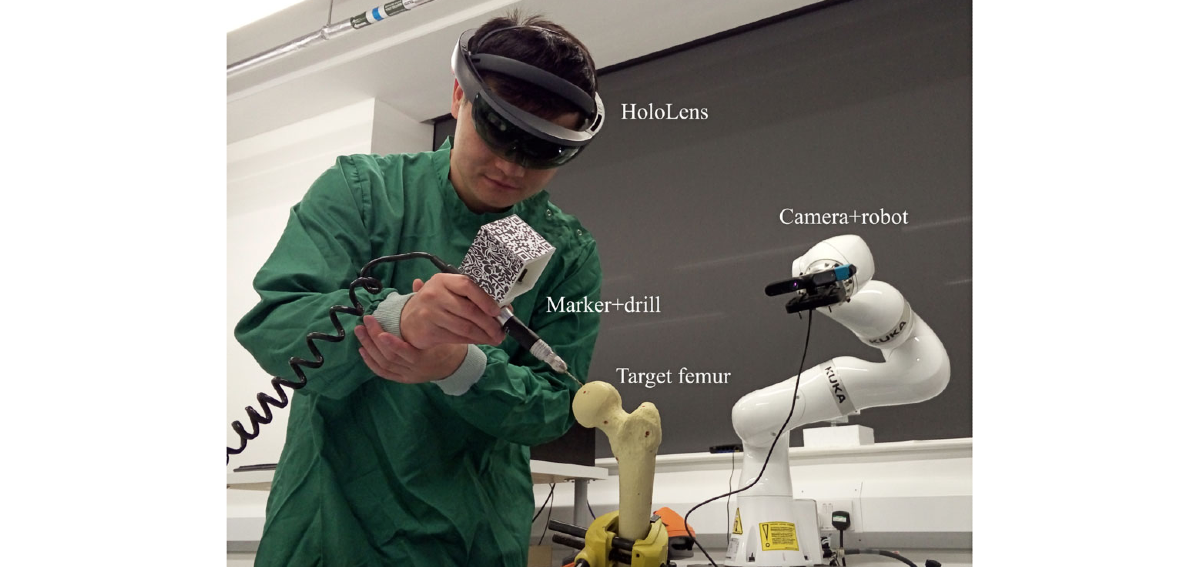
Liu et al [42] developed an augmented reality–based navigation system for hip resurfacing. Reproduced from the cited source which is published under Creative Commons Attribution 4.0 International License [65].

Computer-assisted orthopedic surgery offers obvious advantages for patients, with higher positioning accuracy and fewer outliers; however, its invasiveness, cost, and complexity limit its wide application. To provide seamless computer-aided, improved real-time imaging and a more natural surgical process, Liu et al [42] developed a hip surface replacement navigation system based on AR. Figure 2 shows an AR-based navigation system. To evaluate the accuracy of this navigation system, a pilot hole drilling experiment was conducted using a femoral model. Compared with the preoperative plan, the position and direction of the borehole were found to have an average error of 2 mm and 2°, respectively, and the navigation system was comparable with currently available commercial computer-aided orthopedic systems.

#### Fracture

##### Pelvic Fracture

Percutaneous screw osteosynthesis of pelvic fractures performed under conventional imaging guidance represents a challenge for even experienced surgeons [66]. Befrui et al [43] performed K-wire implantation in long bone phantoms and suprapubic phantoms using a red-green-blue-depth augmented cone-beam CT system and compared K-wire placement performed using AR-based navigation with that performed using conventional C-arm fluoroscopy alone. The results showed that AR navigation significantly reduced the operation time—from 9.9 to 4.1 minutes for long bone phantoms and from 10.9 to 5.5 minutes for suprapubic phantoms. Furthermore, AR-based navigation reduced the intraoperative radiation dose. Finally, the placement accuracy did not significantly differ between the conventional method and the AR method.

Percutaneous sacroiliac screw fixation is a widely accepted method for the treatment of posterior pelvic ring instability [67]. Compared with open reduction and internal fixation, percutaneous sacroiliac screw fixation is associated with less trauma and a lower incidence of postoperative wound infection [52]. The conventional method of achieving accurate screw placement involves the insertion of the screws under fluoroscopic guidance. AR can overlay virtual images onto the real world. Wang et al [44] developed a new sacroiliac screw insertion navigation system based on AR for preoperative planning and evaluated its feasibility and accuracy in cadaveric experiments; see Wang et al [44] for an image of a novel AR-based navigation system. Six complete pelvic specimens were imaged using CT scans, and the pelvis and blood vessels were segmented into 3D models. The ideal trajectory of the sacroiliac screw was designed and visualized as a cylinder. For the intervention, a head-mounted display was used to create a real-time AR environment by superimposing a virtual 3D model onto the surgeon’s field of view. According to the trajectory represented by the cylinder, the screw was drilled into the pelvis. This method was feasible and accurate and may be a valuable tool for assisting percutaneous sacroiliac screw implantation in live surgeries.

In the past decades, the application of computer-aided navigation systems in preoperative planning has greatly reduced surgical risks and improved surgical accuracy [68]. Currently, a few commercial surgical navigation systems have been tested and approved, such as ENLight, NavSuite, Portable Nanostation, and MATRIX POLAR. Augmented reality–based surgical navigation system (AR-SNS) is a surgical navigation system based on AR developed by Chen et al [45] that uses an optical transparent head-mounted display; see Chen et al [45] for an image of an optical see-through head-mounted display. The system includes preoperative surgical planning, registration, and intraoperative tracking. With the help of AR-SNS, surgeons wearing a head-mounted display can view merged images that combine virtual anatomical structures such as soft tissues, blood vessels, and nerves with real scenes during surgery so as to improve the safety and reliability of surgery. AR-SNS can be used to implement the preoperative plan. Percutaneous sacroiliac screw implantation is a very common operation in orthopedics. To avoid damaging important anatomical structures such as the soft tissues, blood vessels, and nerves in the pelvis, a virtual path is created for the surgical drill and rendered on all 2D and 3D views, which improves the accuracy, safety, and reliability of implant surgery.

##### Acetabular Fracture

**Figure 3:**
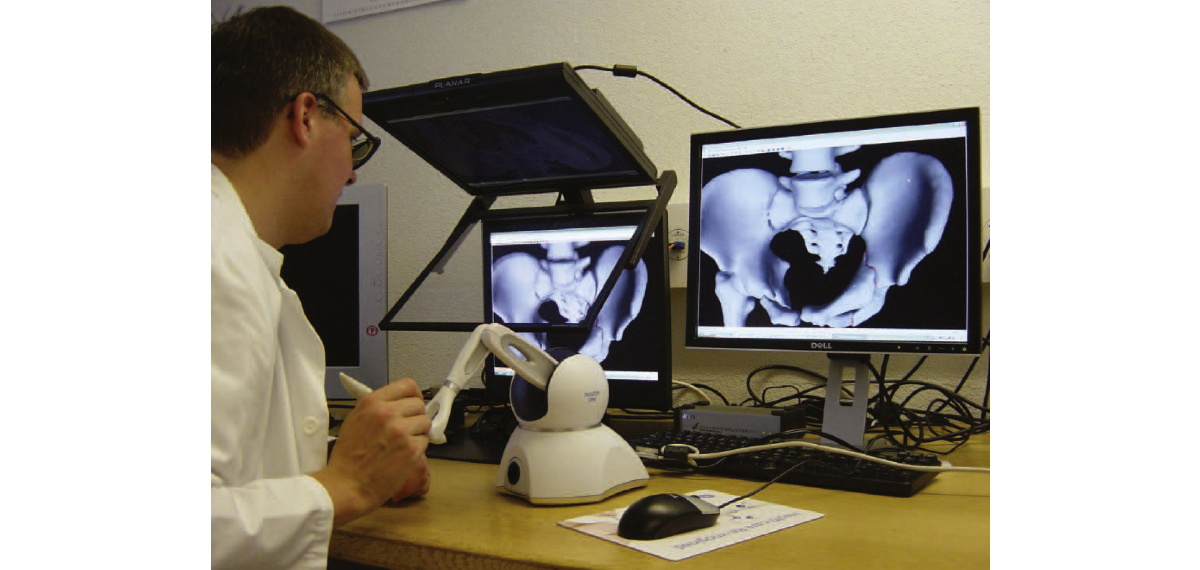
Fornaro et al [46] tested the feasibility of preoperative surgical planning for acetabular fractures. Reproduced from the cited source which is published under Creative Commons Attribution 4.0 International License [65].

Acetabular fractures remain to be one of the most challenging fractures to treat because of the complex anatomy, difficulty in gaining surgical access to the fracture site, and the relatively low incidence of these lesions, resulting in a long learning curve [46,69] Owing of the rarity and complexity of acetabular fractures, experienced acetabular surgeons are needed to conduct specific teaching and learning tasks [69]. Fornaro et al [46] completed an initial study to test the feasibility of preoperative virtual surgical planning in acetabular fractures using a new prototype planning tool based on an interactive AR environment. Figure 3 shows the feasibility of preoperative surgical planning. The software package Amira (version 3.1, TGS, Inc) was used for semiautomatic segmentation of the pelvic bones and fracture fragments. Then, the segmented images were imported into a planning tool (using OpenGL for graphics and the PHANTOM Omni Developer Kit) in the common Standard Triangle Language or Wavefront Object file formats for haptic rendering. The angle and length of the 3D space were measured according to the specific marks visible or accessible on the pelvic bone during the operation. The pelvic surgery prototype planning tool proposed in this study was successfully integrated into the clinical workflow to improve patient-specific preoperative planning and provide visual and tactile information about the injury. The limitation of this study was that the authors did not use current tools to design and simulate interference by soft tissues. Soft-tissue structures, such as muscles and tendons inserted into the pelvic bones, blood vessels, and pelvic organs, were not modeled.

##### Pelvic and Acetabular Fractures

The conventional surgical treatment method for pelvic and acetabular fractures requires complete exposure of the fractures and intraoperative implant contouring after intraoperative fracture reduction so that the reconstruction plate can adapt to the reduced pelvis. This invasive approach often leads to prolonged operation time and considerable injury and bleeding [70]. Shen et al [47] used a special patient-specific AR-assisted preoperative implant design and unilateral pelvic and acetabular surgical contouring system. This system provides a user-friendly interface for simulating fracture reduction and implant design and a low-cost environment for rapid preoperative implant template development. The entire system consists of 2 subsystems: a virtual fracture reduction system and an AR-based auxiliary template system. The surgeon can design the reconstruction plate and its final shape after bending and create a surgical plan for its placement. This results in the development of a digital preoperative implantation model. The final preoperative reconstruction plate is created by mapping the reconstruction plate kit to the virtual plate kit. The software is implemented in C++ under Windows 7 (Microsoft Corp). In conclusion, by using this type of patient-specific implant template for preoperative surgical planning, the process of intraoperative implant contouring is omitted, which minimizes surgical trauma and enables satisfactory reduction and fixation.

##### Femoral Neck Fracture

Extracapsular fractures account for a substantial proportion of femoral neck fractures. Extracapsular femoral neck fractures can be treated using a fixed-angle sliding screw device, which is commonly referred to as a sliding compression or dynamic hip screw. However, the mechanical failure rate is as high as 20% [71-73]. To overcome this, van Duren et al [48] developed a digital fluoroscopic imaging simulator using orthogonal cameras to track colored markers attached to guidewires and thereby create a virtual overlay on fluoroscopic images of the hip. This system was used to calculate the virtual guidewire tip-vertex distance and compare it with the physically measured guidewire tip-vertex distance. This study demonstrated a new AR-based simulation of guidewire insertion in dynamic hip screw surgery. Unlike virtual VR, AR can simulate perspective while allowing students to interact with real instruments and perform operations on bone models.

#### Tumor

**Figure 4:**
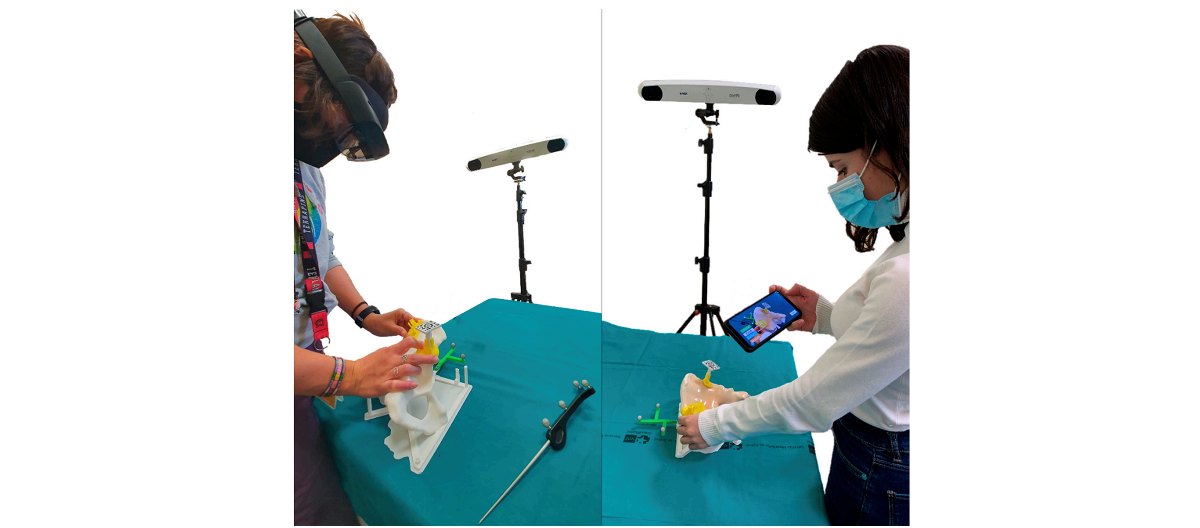
Augmented reality as a tool to guide patient-specific instrumentation placement during pelvic tumor resection by García-Sevilla et al [49]. Reproduced from the cited source which is published under Creative Commons Attribution 4.0 International License [65].

The treatment of pelvic malignancies is a complex scenario for surgeons because in many cases, extensive resection is required, and there is a risk of damage to important structures. During these interventions, accuracy is crucial to minimize local recurrence and maximize limb function. However, when tumors are resected using conventional methods, the probability of obtaining a sufficient resection margin is only 52% [74]. In complex surgical scenarios such as pelvic tumor resection, patient-specific instrument (PSI) has become a valuable osteotomy guidance tool. The accuracy of PSI is similar to that of surgical navigation systems. García-Sevilla et al [49] recommended using AR to guide and verify the placement of PSI. They designed an experiment, using smartphones and HoloLens 2, to evaluate the accuracy of the AR system and compared it with that of conventional apprenticeship. Figure 4 shows a tool for guiding PSI placement. The results showed a significant difference. Their study provided promising results, proving that AR has the potential to easily and effectively overcome the current limitations of PSI, such as the challenges of correct placement, the inability to objectively verify the intervention process, and the possibility that incorrect installation may lead to height deviation from the planned osteotomy height and increase the risk of a positive margin. Pelvic malignancies are often large at the time of diagnosis, and the complex anatomical structure of the pelvis necessitates an accurate osteotomy. Moreover, accuracy is crucial for finding a balance between radical resection and tissue preservation, which is important for obtaining good functional results [75-78]. A recent study of Sawbones models reported that navigation significantly improved the accuracy of osteotomy in pelvic resection compared with manual Sawbones setting [79]. Postl et al [50] evaluated the accuracy of supra-acetabular pelvic tumor resections in human full-body cadavers under realistic operating-room conditions with the help of a navigation system and using K-wires as guidance for the oscillating saw. Under the condition of the simulated operating room, K-wire guidance for supra-acetabular osteotomy was more accurate when using the navigation system than when using freehand osteotomies.

## Discussion

### Principal Findings

The main findings of this study are as follows. First, VAR technology can perform these hip-related surgery steps very well, and the resulting surgery–related complications problems and the probability of poor prognosis of patients are greatly reduced. Second, the application of VR and AR technologies in hip-related preoperative operation simulation and training is perfect for determining the dimensions and the individualization of acetabular and femoral implants so that the most appropriate implants are used for each patient. Moreover, interactive and immersive experience can save time, decrease surgical risk and intraoperative radiation exposure, and reduce the chance of poor outcomes in total hip replacement surgery. Finally, the application of VR and AR technologies in the operating room and during postoperative rehabilitation will become a rapidly developing field in the future.

### Clinical Application of VAR

#### Advantages of VAR in Hip-Related Surgery

VAR technology enables orthopedic surgeons to simulate operations outside the operating room, thereby allowing them to perform repetitive hand movement training in a safe environment. Several simulator models have been developed and studied, including high- and low-fidelity simulators, synthetic bench simulators, animal or human cadaver simulators, and VR simulators. The results of relevant studies [64,80-82] have shown that in the case of orthopedic surgical simulators, the increasing use of virtual models shortens the learning curve for mastering the relevant surgical steps, and 3D computer modeling techniques can help individualize implant design, reduce postoperative complications, and greatly increase patient satisfaction.

A paradigm shift is occurring in the teaching of surgical techniques. The conventional learning mode of apprenticeship training is not efficient and cannot achieve the integration of surgical techniques under varying circumstances. Total hip replacement is critically dependent on the surgeon’s skill and experience, and VR and AR techniques have great potential for rapidly improving the surgical skills required for total hip replacement. Cadaveric training has been the gold standard for surgical training for many years [51,61]. The transition to simulations based on VAR technology began >20 years ago, and currently, training programs based on these technologies play an irreplaceable role in the education of resident surgeons who are just beginning their careers. These training programs break down surgical procedures into tasks and provide gains in surgical technique and expertise in a controlled and relatively safe environment, thereby improving surgical technique and experience. Without causing any harm to patients, students can reach a certain level of surgical skill and accumulate some surgical experience before even entering the operating room.

#### Limitations of VAR in Hip-Related Surgery

However, a potential barrier to the use of VAR technology is the lack of interest in computers among surgeons, who still prefer to use textbooks and journal papers to correct or improve surgical techniques [83]. Therefore, they are more receptive to real-world techniques. Over the past 10 years, an increasing number of studies [84-87] have reported the use of VAR technology for surgical technique training. Training in total hip replacement surgery is demanding and has a steep learning curve. Multiple studies [31,88-91] have highlighted the difficulty less-experienced surgeons face in the simulation of the complex hand movements required during total hip replacement surgery, which can even lead to failure. Therefore, improving trainee acceptance of computer technology is necessary, but simulators involving VAR technology require ongoing follow-up. Current VAR technology simulation training does not simulate soft-tissue dissection, which should be added in the future to provide more productive training. Validation of simulators for surgical training is not difficult to find in the above-mentioned validation pilot studies [39,51,86,87,92]; however, although the validity of the simulators was evaluated, the evaluation criteria used for their effectiveness were often subjective. Although VAR technology simulators appear to have many potential advantages, a recent systematic review [93] of the effectiveness of surgical simulators (virtual, video, and surgical training) based on data from the Australian Safety and Efficacy Register of New Interventional Procedures-Surgical concluded that none of the simulators demonstrated superiority over conventional methods. However, this finding largely reflects a general lack of evidence from the various trials, and it is not corroborated by the results obtained from single randomized controlled trials [51,61,94]. VAR-based surgical simulators have been criticized for their lack of a sense of authentic experience, as after all, they do not involve operations on real bodies. Although many VAR-based simulators are currently available for hip-related surgery, only a few reviews have been published on their use and effects. Most related studies [86,87] in the literature consist of randomized controlled trials that have attempted to validate the use of various existing simulators by means of a modified validity test for more efficient application in clinical teaching tasks.

Simulators are being increasingly used for evaluation and training in clinical learning, and VR and AR techniques are being widely used for procedures such as hip trauma and hip tumor surgeries. Nevertheless, training simulators for total hip replacement have lagged behind those for other surgical procedures. Therefore, creating simulators for procedures related to total hip replacement is warranted.

#### Future Perspectives of VAR in Hip-Related Surgery

##### Ideal Training and Education Simulators Used in VAR

The goal of hip surgery simulation is to improve the operator’s understanding of the anatomy and develop a good touch sensation. Training simulators allow for the repeated practice of surgical techniques before actual surgery, which helps surgeons deepen their visuospatial skills, that is, their visual perception of anatomical structures. Further studies are required to verify whether simulators reduce the odds of poor outcomes and improve the technical ability of trainees to perform actual operations. In addition, more research trials are required to develop simulators for total hip replacement surgery that will shorten the learning curve and increase trainee acceptance. Ideally, the technical skills derived from simulators should be easily transferable to the operating room, thus improving patient satisfaction and helping trainee surgeons accumulate more clinical experience. We believe that the ideal simulator for total hip replacement surgery should be multimodal and provide an immersive environment combining tactile, visual, and auditory cues.

##### AR in Open Surgery Is a Reliable Tool in the Operating Room

AR technology has successfully provided surgeons with a wide range of visual information about anatomical structures and assisted them throughout the operation. AR technology allows surgeons to view the surgical field through a superimposed 3D virtual model of anatomical details. Dennler et al [95] conducted a clinical feasibility study on AR in the operating room. A total of 13 orthopedic surgeons from a Swiss university clinic performed 25 orthopedic surgical procedures wearing HoloLens, a holographic AR headset providing complementary 3D, patient-specific anatomical information. Although the surgeons were generally satisfied with the image quality of the headset device tested here, they also pointed out some technical and ergonomic deficiencies. However, thus far, only a few studies have evaluated the application of AR in the operating room. This is because in open surgery, the registration of virtual and real scenes remains an open problem. AR registration is affected by problems related to organ deformation, out-of-control breathing, and continuous contact between surgical instruments and soft tissue. The application of AR systems and their components in surgery leads to problems and challenges. AR is an effective, reliable, and promising open surgical technique. However, further improvements are required to improve the performance of AR systems and apply them in different operations. To overcome the problems of organ deformation and inaccurate registration, the virtual model must be updated continuously during the operation.

##### VAR Are Universally Used in the Postoperative Rehabilitation of Hip Surgery

After hip trauma or surgery, postoperative rehabilitation is essential to restore damaged functions [96]. Successful treatment requires an appropriate exercise combination and progression to improve joint activity and muscle strengthening and restore physical function [96]. The application of VAR in postoperative remote rehabilitation is attracting the interest of orthopedists. In the past decades, remote virtual rehabilitation gained research interest. With the spread of COVID-19, the role of remote virtual rehabilitation has become even more important [97]. Postoperative rehabilitation is widely performed in neurology. van der Veen et al [98] conducted a pilot study quantifying center-of-mass trajectory during dynamic balance tasks using an HTC Vive tracker fixed to the pelvis. HTC Vive can be used to simulate objects, forces, and interactions between objects with high realism and accuracy. Borglund et al [99] studied feedback from HTC Vive sensors and found that the use of this device resulted in transient performance enhancements in a juggling task in VR. Kayabinar et al [100] studied the effects of VAR-based, robot-assisted gait training on dual-task performance and functional measures in patients with chronic stroke. Blasco et al [101] studied the efficacy of VR tools for physical rehabilitation after total knee replacement. The advantages of VR and AR technologies in postoperative remote rehabilitation have been proven in many medical fields [102]; however, few studies have focused on the use of these technologies for orthopedic rehabilitation [103]. Remote rehabilitation has been proven to be safe and effective. During the COVID-19 pandemic, it ensured telemedicine consultations and greatly reduced the risk of unnecessary travel and physical contact. Extending some aspects of medical practice beyond the physical boundaries of clinical medical facilities is a cutting-edge strategy for meeting growing medical needs. It is also necessary to study the cost-effectiveness of telerehabilitation in the future.

We fully believe that VR has made great progress. With AR technology, simulations of alternative environments have been incorporated into rehabilitation therapy. Remote rehabilitation via virtual technology allows high-quality care to be provided at a low cost. In view of the growing demand for orthopedic rehabilitation and the increasing related costs, VAR technology will be increasingly applied in the physical rehabilitation of patients after hip surgery.

### Limitations

Our systematic review has a few limitations. First, the review lacks a meta-analysis. It was not possible to conduct a meta-analysis because each article involved a different surgical intervention, making the group too heterogeneous. Second, we aimed to cover the use of VR and AR techniques in total hip replacement and reconstruction, hip trauma and fracture, and revision total hip replacement surgery. However, few of the included articles discussed hip tumor surgery simulators.

### Future Research

We expect that in the future, more researchers will apply VR and AR technologies to hip tumor simulators for surgical training and preoperative simulation. The new generation of surgeons should be prepared and willing to adopt these new technologies. Through collaboration among experts in the fields of medicine, engineering, and gaming, we will be able to combine all these areas in the future to fundamentally improve the components of hip surgery.

### Conclusions

This systematic review suggests that VAR technologies have the potential to assist surgeons in performing surgeries faster and more accurately. Although VAR are promising modern technologies, more comparative studies on technical accuracy, operative time, clinical outcomes, and cost-effectiveness are necessary. Moreover, we expect future studies to demonstrate whether augmented technology is beneficial in the field of postoperative rehabilitation.

## Appendix

Multimedia Appendix 1 Overview and definitions of reality technologies.

Multimedia Appendix 2 PRISMA (Preferred Reporting Items for Systematic Reviews and Meta-Analyses) 2020 checklist.

Multimedia Appendix 3 Joanna Briggs Institute Critical Appraisal Checklist for case report and series studies.

Multimedia Appendix 4 Quality appraisal for cadaveric studies checklist.

## Abbreviations

AR: augmented reality
AR-SNS: augmented reality–based surgical navigation system
CT: computed tomography
PRISMA: Preferred Reporting Items for Systematic Reviews and Meta‐Analyses
PSI: patient-specific instrument
VAR: virtual and augmented reality
VR: virtual reality

## Data Availability

All data generated or analyzed during this study are included in the published article. All figures have been published in an open access journal.
