# Supplementary material for: Application of Virtual and Augmented Reality Technology in Hip Surgery: Systematic Review
Source: J Med Internet Res. 2023 Mar 10;25:e37599. doi: 10.2196/37599 (PMC10039409; doi:10.2196/37599)
Supplement: Multimedia Appendix 1 [file jmir_v25i1e37599_app1.docx]

**Table 1. Overview and definitions of reality technologies**

| **Classification** | **Explanation** |
| --- | --- |
| Virtual reality | The user is completely immersed in an artificial, computer-generated environment. The system can also produce artificial sounds and other stimuli.  Applications in hip surgery are limited to preoperative planning, patient education, and resident training. |
| Augmented reality | The digital display is superimposed on the surface of the real world, allowing depth perception.  At present, it can be used in smart phones and head-mounted display platforms for preoperative planning, intraoperative guidance, and resident training. |
