# Supplementary material for: Application of Virtual and Augmented Reality Technology in Hip Surgery: Systematic Review
Source: J Med Internet Res. 2023 Mar 10;25:e37599. doi: 10.2196/37599 (PMC10039409; doi:10.2196/37599)
Supplement: Multimedia Appendix 3 [file jmir_v25i1e37599_app3.docx]

| JOANNA BRIGGS INSTITUTE CRITICAL APPRAISAL TOOL FOR CASE REPORT STUDIES  Yes = 2 Unclear = 1 No = 0 | | | | | | | | | | |
| --- | --- | --- | --- | --- | --- | --- | --- | --- | --- | --- |
| Study | 1. Were patient’s demographic characteristics clearly described? | 2. Was the patient’s history clearly described and presented as a timeline? | 3. Was the current clinical condition of the patient on presentation clearly described? | 4. Were diagnostic tests or assessment methods and the results clearly described? | 5. Was the intervention(s) or treatment procedure(s) clearly described? | 6. Was the post-intervention clinical condition clearly described? | 7. Were adverse events (harms) or unanticipated events identified and described? | 8. Does the case report provide takeaway lessons? | Total score | % |
| Cimerman et al[25] | 2 | 2 | 0 | 1 | 2 | 2 | 2 | 2 | 13/16 | 81% |

| JOANNA BRIGGS INSTITUTE CRITICAL APPRAISAL TOOL FOR CASE SERIES STUDIES  Yes = 2 Unclear = 1 No = 0 | | | | | | | | | | | | |
| --- | --- | --- | --- | --- | --- | --- | --- | --- | --- | --- | --- | --- |
| Study | 1. Were there clear criteria for inclusion in the case series? | 2. Was the condition measured in a standard, reliable way for all participants included in the case series? | 3. Were valid methods used for identification of the condition for all participants included in the case series? | 4. Did the case series have consecutive inclusion of participants? | 5. Did the case series have complete inclusion of participants? | 6. Was there clear reporting of the demographics of the participants in the study? | 7. Was there clear reporting of clinical information of the participants? | 8. Were the outcomes or follow up results of cases clearly reported? | 9. Was there clear reporting of the presenting site(s)/clinic(s) demographic information? | 10. Was statistical analysis appropriate? | TOTAL | % |
| Sato et al[17] | 1 | 2 | 2 | 2 | 2 | 2 | 2 | 2 | 2 | 1 | 18/20 | 90 |
| Digioia et al[18] | 2 | 2 | 1 | 2 | 1 | 2 | 2 | 2 | 2 | 2 | 18/20 | 90 |
| Takada et al[19] | 2 | 2 | 2 | 2 | 2 | 2 | 2 | 2 | 2 | 2 | 20/20 | 100 |
| Brouwers et al[26] | 2 | 2 | 2 | 2 | 2 | 2 | 2 | 2 | 2 | 2 | 20/20 | 100 |
| Blyth et al[28] | 2 | 2 | 2 | 2 | 2 | 0 | 0 | 0 | 1 | 2 | 13/20 | 65 |
| Rambani et al[30] | 2 | 2 | 1 | 2 | 2 | 2 | 1 | 2 | 2 | 2 | 18/20 | 90 |
| Khanduja et al[33] | 2 | 2 | 1 | 2 | 2 | 2 | 1 | 2 | 2 | 2 | 18/20 | 90 |
| Bishop et al[34] | 1 | 1 | 2 | 2 | 2 | 2 | 2 | 2 | 2 | 2 | 18/20 | 90 |
| Fascio et al[36] | 2 | 1 | 2 | 2 | 2 | 2 | 2 | 2 | 2 | 2 | 19/20 | 95 |
| Alexander et al[38] | 2 | 1 | 1 | 1 | 2 | 2 | 1 | 2 | 2 | 2 | 16/20 | 80 |
| Ogawa et al[39] | 1 | 1 | 1 | 2 | 2 | 1 | 2 | 2 | 2 | 2 | 16/20 | 80 |
| Logishetty et al[41] | 2 | 2 | 2 | 2 | 2 | 2 | 2 | 2 | 2 | 2 | 20/20 | 100 |
| García-Sevilla et al[49] | 2 | 1 | 2 | 2 | 2 | 1 | 1 | 1 | 1 | 2 | 15/20 | 75 |
| Chen et al[45] | 2 | 1 | 1 | 1 | 1 | 2 | 2 | 2 | 2 | 2 | 16/20 | 80 |
| Bartlett et al[21] | 1 | 2 | 2 | 2 | 2 | 2 | 2 | 2 | 2 | 2 | 19/20 | 98 |
| Racy et al[31] | 2 | 1 | 2 | 2 | 2 | 2 | 2 | 2 | 2 | 2 | 19/20 | 95 |
