# Supplementary material for: Application of Virtual and Augmented Reality Technology in Hip Surgery: Systematic Review
Source: J Med Internet Res. 2023 Mar 10;25:e37599. doi: 10.2196/37599 (PMC10039409; doi:10.2196/37599)
Supplement: Multimedia Appendix 4 [file jmir_v25i1e37599_app4.docx]

**Multimedia Appendix 4.** Quality appraisal for cadaveric studies checklist.

| QUACS (Quality Appraisal for Cadaveric Studies)  Yes = 1 No = 0 | | |
| --- | --- | --- |
| **Item** | Wang et al [44] | Postl et al [50] |
| Objective stated | 1 | 1 |
| Basic information about sample is included | 1 | 1 |
| Applied methods are described comprehensibly | 1 | 1 |
| Study reports condition of the examined specimens | 1 | 0 |
| Education of dissecting researchers is stated | 0 | 1 |
| Findings are observed by more than one researcher | 1 | 1 |
| Results presented thoroughly and precise | 1 | 1 |
| Statistical methods appropriate | 0 | 1 |
| Details about consistency of findings are given | 0 | 1 |
| Photographs of the observations are included | 1 | 1 |
| Study is discussed within the context of the current evidence | 1 | 1 |
| Clinical implications of the results are discussed | 1 | 1 |
| Limitations of the study are addressed | 1 | 1 |
| Totals (%) | 11/13 (85%) | 12/13 (92%) |
